# 5-Bromo-3-ethyl­sulfinyl-2-(4-methyl­phen­yl)-1-benzo­furan

**DOI:** 10.1107/S1600536814014470

**Published:** 2014-06-25

**Authors:** Hong Dae Choi, Pil Ja Seo, Uk Lee

**Affiliations:** a Department of Chemistry, Dongeui University, San 24 Kaya-dong, Busanjin-gu, Busan 614-714, Republic of Korea; b Department of Chemistry, Pukyong National University, 599-1 Daeyeon 3-dong, Nam-gu, Busan 608-737, Republic of Korea

**Keywords:** crystal structure

## Abstract

In the title compound, C_17_H_15_BrO_2_S, the dihedral angle between the plane of the benzo­furan ring system [r.m.s. deviation = 0.004 (3) Å] and that of the 4-methyl­phenyl ring is 0.9 (2)°. In the crystal, mol­ecules are linked by C—H⋯O, C—H⋯π and Br⋯π [3.636 (2) Å] inter­actions, and by π–π inter­actions between the 4-methyl­phenyl and furan rings of neighbouring mol­ecules [centroid–centroid distance = 3.650 (2) Å], forming a three-dimensional network.

## Related literature   

For background information and the crystal structures of related compounds, see: Choi *et al.* (2010 ▶, 2012 ▶).
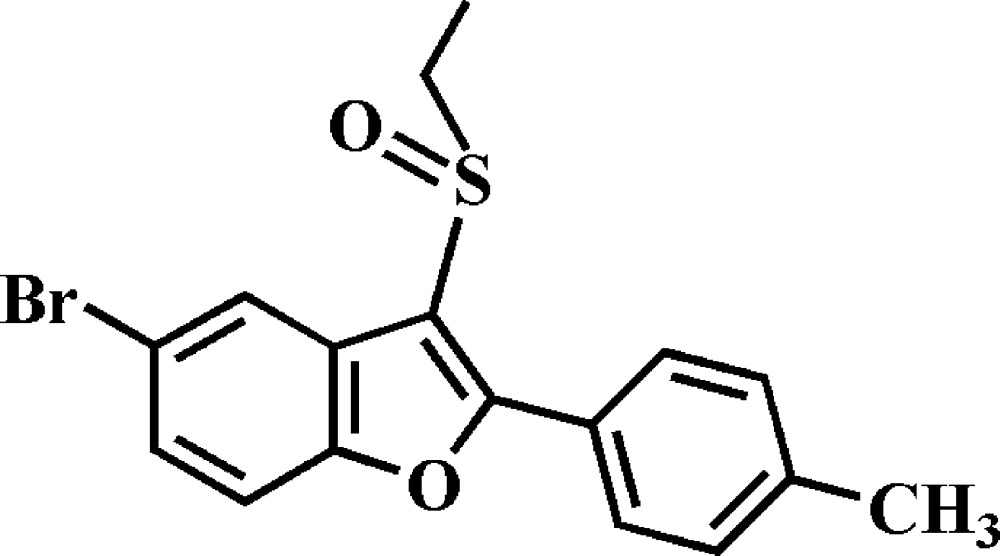


## Experimental   

### 

#### Crystal data  

C_17_H_15_BrO_2_S
*M* *_r_* = 363.26
Monoclinic, 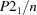

*a* = 5.0202 (3) Å
*b* = 25.0471 (12) Å
*c* = 12.2430 (6) Å
β = 100.340 (2)°
*V* = 1514.45 (14) Å^3^
*Z* = 4
Mo *K*α radiation
μ = 2.85 mm^−1^
*T* = 173 K
0.60 × 0.14 × 0.12 mm

#### Data collection  

Bruker SMART APEXII CCD diffractometer
Absorption correction: multi-scan (*SADABS*; Bruker, 2009 ▶) *T*_min_ = 0.541, *T*_max_ = 0.746
26373 measured reflections
3868 independent reflections
3104 reflections with *I* > 2σ(*I*)
*R*_int_ = 0.058

#### Refinement  

*R*[*F* ^2^ > 2σ(*F* ^2^)] = 0.052
*wR*(*F* ^2^) = 0.139
*S* = 1.06
3868 reflections
192 parameters
H-atom parameters constrained
Δρ_max_ = 1.97 e Å^−3^
Δρ_min_ = −0.83 e Å^−3^

### 

Data collection: *APEX2* (Bruker, 2009 ▶); cell refinement: *SAINT* (Bruker, 2009 ▶); data reduction: *SAINT*; program(s) used to solve structure: *SHELXS97* (Sheldrick, 2008 ▶); program(s) used to refine structure: *SHELXL97* (Sheldrick, 2008 ▶); molecular graphics: *ORTEP-3 for Windows* (Farrugia, 2012 ▶) and *DIAMOND* (Brandenburg, 1998 ▶); software used to prepare material for publication: *SHELXS97*.

## Supplementary Material

Crystal structure: contains datablock(s) I. DOI: 10.1107/S1600536814014470/ff2130sup1.cif

Structure factors: contains datablock(s) I. DOI: 10.1107/S1600536814014470/ff2130Isup2.hkl

Supporting information file. DOI: 10.1107/S1600536814014470/ff2130Isup3.cml

CCDC reference: 1009166

Additional supporting information:  crystallographic information; 3D view; checkCIF report

## Figures and Tables

**Table 1:** Hydrogen-bond geometry (Å, °) *Cg*1 is the centroid of the C9–C14 4-methyl­phenyl ring.

*D*—H⋯*A*	*D*—H	H⋯*A*	*D*⋯*A*	*D*—H⋯*A*
C13—H13⋯O2^i^	0.95	2.58	3.507 (4)	164
C16—H16*B*⋯O2^ii^	0.99	2.26	3.110 (5)	144
C15—H15*C*⋯*Cg*1^ii^	0.99	2.78	3.607 (3)	143

Symmetry codes: (i) 
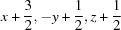
; (ii) 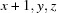.

**Fig. 1.**
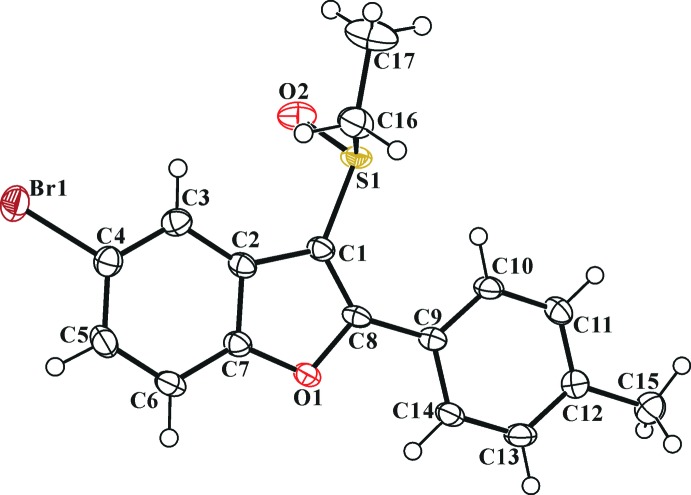
The molecular structure of the title molecule with the atom numbering scheme. The displacement ellipsoids are drawn at the 50% probability level. The hydrogen atoms are presented as small spheres of arbitrary radius.

**Fig. 2.**
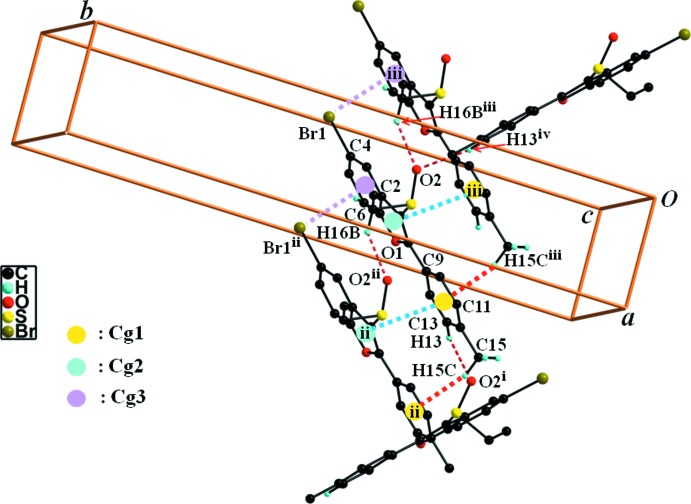
A view of the C—H···O, C—H···π, Br···π and π···π interactions (dotted lines) in the crystal structure of the title compound. H atoms non-participating in hydrogen-bonding were omitted for clarity. [Symmetry codes: (i) x + 3/2, - y + 1/2, z + 1/2; (ii) x + 1, y, z; (iii) x - 1, y, z; (iv) x - 3/2, - y + 1/2, z - 1/2.]

## supplementary crystallographic information

### S1. Comment

As a part of our ongoing project of 2-aryl-5-bromo-1-benzofuran derivatives containing [3-ethylsulfinyl-2-(4-fluorophenyl)] (Choi *et al.*, 2010) and [2-(4-methylphenyl)-3-methylsulfinyl] (Choi *et al.*, 2012) substituents. We report herein on the crystal structure of the title compound.

In the title molecule (Fig. 1), the benzofuran unit is essentially planar, with a mean deviation of 0.004 (3) Å from the least-squares plane defined by the nine constituent atoms. The 4-methylphenyl ring is essentially planar, with a mean deviation of 0.002 (3) Å from the least-squares plane defined by the six constituent atoms. The dihedral angle formed by the benzofuran ring system and the 4-methylphenyl ring is 0.9 (2)°. In the crystal structure (Fig. 2), molecules are linked by C—H···O and C—H···π hydrogen bonds (Table 1, Cg1 is the centroid of the C9–C14 4-methylphenyl ring) and C4—Br1···π interactions between the bromine atom and the benzene ring of a neighbouring molecule with a Br1···Cg3^iii^ = 3.636 (2) Å (Cg3 is the centroid of the C2–C7 benzene ring). The crystal packing (Fig. 2) also exhibits π···π interactions between the 4-methylphenyl and furan rings of neighbouring molecules, with a Cg1···Cg2^ii^ distance of 3.650 (2) Å (Cg2 is the centroid of the C1/C2/C7/O1/C8 furan ring), forming a three-dimensional network.

### S2. Experimental

3-Chloroperoxybenzoic acid (77%, 269 mg, 1.2 mmol) was added in small portions to a stirred solution of 5-bromo-3-ethylsulfanyl-2-(4-methylphenyl)-1-benzofuran (382 mg, 1.1 mmol) in dichloromethane (35 mL) at 273 K. After being stirred at room temperature for 5h, the mixture was washed with saturated sodium bicarbonate solution and the organic layer was separated, dried over magnesium sulfate, filtered and concentrated at reduced pressure. The residue was purified by column chromatography (hexane–ethyl acetate, 1:1 *v*/*v*) to afford the title compound as a colorless solid [yield 71%, m.p. 404–405 K; *R*_f_ = 0.54 (hexane–ethyl acetate, 1:1 *v*/*v*)]. Single crystals suitable for X-ray diffraction were prepared by slow evaporation of a solution of the title compound in benzene at room temperature.

### S3. Refinement

All H atoms were positioned geometrically and refined using a riding model, with C—H = 0.95 Å for aryl, 0.99 Å for methylene and 0.98 Å for methyl H atoms, respectively. *U*_iso_ (H) = 1.2*U*_eq_ (C) for aryl and methylene, and 1.5*U*_eq_ (C) for methyl H atoms. The positions of methyl hydrogens were optimized using the SHELXL-97's command AFIX 137 (Sheldrick, 2008). The highest peak in the difference map is 0.91 Å from Br1 and the largest hole is 0.59 Å from Br1.

### Crystal data

**Table:**

C_17_H_15_BrO_2_S	*F*(000) = 736
*M**_r_* = 363.26	*D*_x_ = 1.593 Mg m^−^^3^
Monoclinic, *P*2_1_/*n*	Melting point = 405–404 K
Hall symbol: -P 2yn	Mo *K*α radiation, λ = 0.71073 Å
*a* = 5.0202 (3) Å	Cell parameters from 7637 reflections
*b* = 25.0471 (12) Å	θ = 2.4–27.2°
*c* = 12.2430 (6) Å	µ = 2.85 mm^−^^1^
β = 100.340 (2)°	*T* = 173 K
*V* = 1514.45 (14) Å^3^	Block, colourless
*Z* = 4	0.60 × 0.14 × 0.12 mm

### Data collection

**Table:**

Bruker SMART APEXII CCD diffractometer	3868 independent reflections
Radiation source: rotating anode	3104 reflections with *I* > 2σ(*I*)
Graphite multilayer monochromator	*R*_int_ = 0.058
Detector resolution: 10.0 pixels mm^-1^	θ_max_ = 28.6°, θ_min_ = 1.6°
φ and ω scans	*h* = −6→6
Absorption correction: multi-scan (*SADABS*; Bruker, 2009)	*k* = −33→32
*T*_min_ = 0.541, *T*_max_ = 0.746	*l* = −16→16
26373 measured reflections	

### Refinement

**Table:**

Refinement on *F*^2^	Primary atom site location: structure-invariant direct methods
Least-squares matrix: full	Secondary atom site location: difference Fourier map
*R*[*F*^2^ > 2σ(*F*^2^)] = 0.052	Hydrogen site location: difference Fourier map
*wR*(*F*^2^) = 0.139	H-atom parameters constrained
*S* = 1.06	*w* = 1/[σ^2^(*F*_o_^2^) + (0.0648*P*)^2^ + 3.2187*P*] where *P* = (*F*_o_^2^ + 2*F*_c_^2^)/3
3868 reflections	(Δ/σ)_max_ = 0.002
192 parameters	Δρ_max_ = 1.97 e Å^−^^3^
0 restraints	Δρ_min_ = −0.83 e Å^−^^3^

### Special details

**Table:**

Geometry. All esds (except the esd in the dihedral angle between two l.s. planes) are estimated using the full covariance matrix. The cell esds are taken into account individually in the estimation of esds in distances, angles and torsion angles; correlations between esds in cell parameters are only used when they are defined by crystal symmetry. An approximate (isotropic) treatment of cell esds is used for estimating esds involving l.s. planes.
Refinement. Refinement of F^2^ against ALL reflections. The weighted R-factor wR and goodness of fit S are based on F^2^, conventional R-factors R are based on F, with F set to zero for negative F^2^. The threshold expression of F^2^ > 2sigma(F^2^) is used only for calculating R-factors(gt) etc. and is not relevant to the choice of reflections for refinement. R-factors based on F^2^ are statistically about twice as large as those based on F, and R- factors based on ALL data will be even larger.

### Fractional atomic coordinates and isotropic or equivalent isotropic displacement parameters (Å^2^)

**Table:**

	*x*	*y*	*z*	*U*_iso_*/*U*_eq_	
Br1	−0.00958 (8)	0.504511 (15)	0.82039 (3)	0.03501 (14)	
S1	0.56805 (19)	0.35409 (3)	0.54542 (7)	0.0275 (2)	
O1	0.8410 (5)	0.33583 (9)	0.86960 (18)	0.0242 (5)	
O2	0.2710 (5)	0.36245 (11)	0.5189 (2)	0.0368 (6)	
C1	0.6586 (7)	0.35476 (13)	0.6920 (3)	0.0229 (6)	
C2	0.5277 (7)	0.38802 (13)	0.7630 (3)	0.0220 (6)	
C3	0.3264 (7)	0.42681 (13)	0.7465 (3)	0.0258 (7)	
H3	0.2391	0.4368	0.6740	0.031*	
C4	0.2582 (7)	0.45033 (14)	0.8400 (3)	0.0266 (7)	
C5	0.3796 (8)	0.43619 (15)	0.9476 (3)	0.0308 (8)	
H5	0.3257	0.4532	1.0095	0.037*	
C6	0.5790 (8)	0.39732 (15)	0.9641 (3)	0.0304 (8)	
H6	0.6644	0.3869	1.0366	0.037*	
C7	0.6481 (7)	0.37435 (13)	0.8707 (3)	0.0236 (7)	
C8	0.8441 (7)	0.32375 (12)	0.7603 (2)	0.0208 (6)	
C9	1.0400 (7)	0.28307 (13)	0.7429 (3)	0.0214 (6)	
C10	1.0737 (7)	0.26723 (14)	0.6372 (3)	0.0253 (7)	
H10	0.9656	0.2830	0.5738	0.030*	
C11	1.2624 (7)	0.22877 (14)	0.6235 (3)	0.0272 (7)	
H11	1.2815	0.2185	0.5505	0.033*	
C12	1.4238 (7)	0.20489 (13)	0.7136 (3)	0.0250 (7)	
C13	1.3907 (8)	0.22062 (15)	0.8188 (3)	0.0304 (8)	
H13	1.4991	0.2045	0.8817	0.037*	
C14	1.2043 (8)	0.25910 (14)	0.8346 (3)	0.0282 (7)	
H14	1.1873	0.2694	0.9077	0.034*	
C15	1.6299 (8)	0.16284 (15)	0.6982 (3)	0.0326 (8)	
H15A	1.6346	0.1589	0.6189	0.049*	
H15B	1.5796	0.1287	0.7280	0.049*	
H15C	1.8091	0.1737	0.7376	0.049*	
C16	0.7154 (8)	0.41740 (17)	0.5188 (3)	0.0343 (8)	
H16A	0.6577	0.4453	0.5669	0.041*	
H16B	0.9154	0.4149	0.5359	0.041*	
C17	0.6247 (12)	0.4323 (2)	0.3983 (4)	0.0561 (13)	
H17A	0.6821	0.4046	0.3510	0.084*	
H17B	0.7062	0.4665	0.3835	0.084*	
H17C	0.4270	0.4355	0.3822	0.084*	

### Atomic displacement parameters (Å^2^)

**Table:**

	*U*^11^	*U*^22^	*U*^33^	*U*^12^	*U*^13^	*U*^23^
Br1	0.0368 (2)	0.0297 (2)	0.0398 (2)	0.00835 (15)	0.01023 (16)	−0.00114 (15)
S1	0.0371 (5)	0.0267 (4)	0.0158 (4)	0.0048 (3)	−0.0031 (3)	−0.0012 (3)
O1	0.0324 (13)	0.0245 (12)	0.0153 (10)	0.0040 (9)	0.0033 (9)	−0.0006 (9)
O2	0.0348 (15)	0.0414 (16)	0.0300 (13)	−0.0066 (12)	−0.0056 (11)	0.0023 (12)
C1	0.0293 (17)	0.0211 (15)	0.0174 (14)	0.0008 (12)	0.0019 (12)	−0.0020 (12)
C2	0.0274 (16)	0.0197 (15)	0.0181 (14)	−0.0028 (12)	0.0021 (12)	−0.0032 (12)
C3	0.0268 (16)	0.0233 (16)	0.0257 (16)	−0.0008 (13)	0.0005 (13)	−0.0001 (13)
C4	0.0255 (16)	0.0254 (16)	0.0289 (17)	−0.0001 (13)	0.0051 (13)	−0.0010 (13)
C5	0.041 (2)	0.0293 (18)	0.0246 (17)	0.0030 (15)	0.0126 (15)	−0.0033 (14)
C6	0.040 (2)	0.0336 (19)	0.0170 (15)	0.0051 (15)	0.0032 (14)	−0.0002 (13)
C7	0.0298 (17)	0.0207 (15)	0.0195 (15)	0.0009 (13)	0.0022 (13)	−0.0016 (12)
C8	0.0287 (16)	0.0192 (14)	0.0134 (13)	−0.0034 (12)	0.0004 (11)	−0.0011 (11)
C9	0.0266 (16)	0.0194 (14)	0.0172 (14)	−0.0023 (12)	0.0015 (12)	0.0001 (11)
C10	0.0318 (17)	0.0253 (16)	0.0178 (15)	0.0022 (13)	0.0021 (13)	0.0045 (12)
C11	0.0346 (18)	0.0261 (17)	0.0215 (16)	−0.0004 (14)	0.0069 (13)	−0.0033 (13)
C12	0.0262 (16)	0.0223 (16)	0.0265 (17)	−0.0013 (13)	0.0048 (13)	−0.0014 (13)
C13	0.0362 (19)	0.0324 (19)	0.0203 (16)	0.0053 (15)	−0.0014 (14)	0.0046 (14)
C14	0.0377 (19)	0.0297 (18)	0.0164 (15)	0.0064 (14)	0.0024 (13)	−0.0010 (13)
C15	0.0338 (19)	0.0272 (18)	0.037 (2)	0.0016 (15)	0.0054 (15)	−0.0017 (15)
C16	0.0335 (19)	0.042 (2)	0.0271 (18)	−0.0040 (16)	0.0052 (15)	0.0024 (16)
C17	0.079 (4)	0.056 (3)	0.032 (2)	−0.011 (3)	0.004 (2)	0.016 (2)

### Geometric parameters (Å, º)

**Table:**

Br1—C4	1.895 (4)	C9—C14	1.403 (4)
S1—O2	1.483 (3)	C10—C11	1.382 (5)
S1—C1	1.770 (3)	C10—H10	0.9500
S1—C16	1.804 (4)	C11—C12	1.383 (5)
O1—C7	1.369 (4)	C11—H11	0.9500
O1—C8	1.375 (4)	C12—C13	1.384 (5)
C1—C8	1.375 (4)	C12—C15	1.512 (5)
C1—C2	1.445 (4)	C13—C14	1.381 (5)
C2—C3	1.390 (5)	C13—H13	0.9500
C2—C7	1.391 (4)	C14—H14	0.9500
C3—C4	1.383 (5)	C15—H15A	0.9800
C3—H3	0.9500	C15—H15B	0.9800
C4—C5	1.395 (5)	C15—H15C	0.9800
C5—C6	1.385 (5)	C16—C17	1.511 (6)
C5—H5	0.9500	C16—H16A	0.9900
C6—C7	1.379 (5)	C16—H16B	0.9900
C6—H6	0.9500	C17—H17A	0.9800
C8—C9	1.458 (5)	C17—H17B	0.9800
C9—C10	1.392 (5)	C17—H17C	0.9800
			
O2—S1—C1	106.61 (16)	C11—C10—H10	119.6
O2—S1—C16	105.37 (17)	C9—C10—H10	119.6
C1—S1—C16	97.81 (17)	C10—C11—C12	121.4 (3)
C7—O1—C8	107.2 (2)	C10—C11—H11	119.3
C8—C1—C2	106.9 (3)	C12—C11—H11	119.3
C8—C1—S1	129.3 (3)	C11—C12—C13	117.9 (3)
C2—C1—S1	123.6 (2)	C11—C12—C15	121.2 (3)
C3—C2—C7	119.4 (3)	C13—C12—C15	120.9 (3)
C3—C2—C1	135.5 (3)	C14—C13—C12	121.8 (3)
C7—C2—C1	105.1 (3)	C14—C13—H13	119.1
C4—C3—C2	117.3 (3)	C12—C13—H13	119.1
C4—C3—H3	121.3	C13—C14—C9	120.1 (3)
C2—C3—H3	121.3	C13—C14—H14	119.9
C3—C4—C5	122.8 (3)	C9—C14—H14	119.9
C3—C4—Br1	118.4 (3)	C12—C15—H15A	109.5
C5—C4—Br1	118.7 (3)	C12—C15—H15B	109.5
C6—C5—C4	119.8 (3)	H15A—C15—H15B	109.5
C6—C5—H5	120.1	C12—C15—H15C	109.5
C4—C5—H5	120.1	H15A—C15—H15C	109.5
C7—C6—C5	117.1 (3)	H15B—C15—H15C	109.5
C7—C6—H6	121.5	C17—C16—S1	109.4 (3)
C5—C6—H6	121.5	C17—C16—H16A	109.8
O1—C7—C6	125.8 (3)	S1—C16—H16A	109.8
O1—C7—C2	110.6 (3)	C17—C16—H16B	109.8
C6—C7—C2	123.5 (3)	S1—C16—H16B	109.8
C1—C8—O1	110.1 (3)	H16A—C16—H16B	108.2
C1—C8—C9	135.0 (3)	C16—C17—H17A	109.5
O1—C8—C9	114.9 (3)	C16—C17—H17B	109.5
C10—C9—C14	118.0 (3)	H17A—C17—H17B	109.5
C10—C9—C8	122.2 (3)	C16—C17—H17C	109.5
C14—C9—C8	119.8 (3)	H17A—C17—H17C	109.5
C11—C10—C9	120.8 (3)	H17B—C17—H17C	109.5
			
O2—S1—C1—C8	141.1 (3)	C2—C1—C8—O1	−0.8 (4)
C16—S1—C1—C8	−110.3 (3)	S1—C1—C8—O1	−177.1 (2)
O2—S1—C1—C2	−34.7 (3)	C2—C1—C8—C9	−179.4 (3)
C16—S1—C1—C2	74.0 (3)	S1—C1—C8—C9	4.3 (6)
C8—C1—C2—C3	−179.6 (4)	C7—O1—C8—C1	1.0 (4)
S1—C1—C2—C3	−3.0 (6)	C7—O1—C8—C9	179.9 (3)
C8—C1—C2—C7	0.3 (4)	C1—C8—C9—C10	0.2 (6)
S1—C1—C2—C7	176.9 (3)	O1—C8—C9—C10	−178.3 (3)
C7—C2—C3—C4	0.6 (5)	C1—C8—C9—C14	179.4 (4)
C1—C2—C3—C4	−179.5 (4)	O1—C8—C9—C14	0.8 (4)
C2—C3—C4—C5	−0.9 (5)	C14—C9—C10—C11	0.4 (5)
C2—C3—C4—Br1	178.6 (2)	C8—C9—C10—C11	179.6 (3)
C3—C4—C5—C6	0.5 (6)	C9—C10—C11—C12	−0.1 (5)
Br1—C4—C5—C6	−179.0 (3)	C10—C11—C12—C13	0.1 (5)
C4—C5—C6—C7	0.2 (6)	C10—C11—C12—C15	180.0 (3)
C8—O1—C7—C6	179.5 (3)	C11—C12—C13—C14	−0.4 (6)
C8—O1—C7—C2	−0.8 (4)	C15—C12—C13—C14	179.8 (4)
C5—C6—C7—O1	179.3 (3)	C12—C13—C14—C9	0.7 (6)
C5—C6—C7—C2	−0.4 (6)	C10—C9—C14—C13	−0.7 (5)
C3—C2—C7—O1	−179.8 (3)	C8—C9—C14—C13	−179.9 (3)
C1—C2—C7—O1	0.3 (4)	O2—S1—C16—C17	−60.2 (4)
C3—C2—C7—C6	0.0 (5)	C1—S1—C16—C17	−169.9 (3)
C1—C2—C7—C6	−179.9 (3)		

### Hydrogen-bond geometry (Å, º)

Cg1 is the centroid of the C9–C14 4-methylphenyl ring.

**Table:**

*D*—H···*A*	*D*—H	H···*A*	*D*···*A*	*D*—H···*A*
C13—H13···O2^i^	0.95	2.58	3.507 (4)	164
C16—H16*B*···O2^ii^	0.99	2.26	3.110 (5)	144
C15—H15*C*···*Cg*1^ii^	0.99	2.78	3.607 (3)	143

Symmetry codes: (i) *x*+3/2, −*y*+1/2, *z*+1/2; (ii) *x*+1, *y*, *z*.
